# Audiovisual Emotional Congruency Modulates the Stimulus-Driven Cross-Modal Spread of Attention

**DOI:** 10.3390/brainsci12091229

**Published:** 2022-09-10

**Authors:** Minran Chen, Song Zhao, Jiaqi Yu, Xuechen Leng, Mengdie Zhai, Chengzhi Feng, Wenfeng Feng

**Affiliations:** 1 Department of Psychology, School of Education, Soochow University, Suzhou 215123, China; 2 Research Center for Psychology and Behavioral Sciences, Soochow University, Suzhou 215123, China

**Keywords:** emotional congruency, spread of attention, stimulus-driven, cross-modal interaction, ERP

## Abstract

It has been reported that attending to stimuli in visual modality can spread to task-irrelevant but synchronously presented stimuli in auditory modality, a phenomenon termed the cross-modal spread of attention, which could be either stimulus-driven or representation-driven depending on whether the visual constituent of an audiovisual object is further selected based on the object representation. The stimulus-driven spread of attention occurs whenever a task-irrelevant sound synchronizes with an attended visual stimulus, regardless of the cross-modal semantic congruency. The present study recorded event-related potentials (ERPs) to investigate whether the stimulus-driven cross-modal spread of attention could be modulated by audio-visual emotional congruency in a visual oddball task where emotion (positive/negative) was task-irrelevant. The results first demonstrated a prominent stimulus-driven spread of attention regardless of audio-visual emotional congruency by showing that for all audiovisual pairs, the extracted ERPs to the auditory constituents of audiovisual stimuli within the time window of 200–300 ms were significantly larger than ERPs to the same auditory stimuli delivered alone. However, the amplitude of this stimulus-driven auditory Nd component during 200–300 ms was significantly larger for emotionally incongruent than congruent audiovisual stimuli when their visual constituents’ emotional valences were negative. Moreover, the Nd was sustained during 300–400 ms only for the incongruent audiovisual stimuli with emotionally negative visual constituents. These findings suggest that although the occurrence of the stimulus-driven cross-modal spread of attention is independent of audio-visual emotional congruency, its magnitude is nevertheless modulated even when emotion is task-irrelevant.

## 1. Introduction

In recent years, researchers have increasingly focused on the neurophysiological mechanisms involved in multisensory processing. It has long been a question how the brain integrates information from different modalities to create the perception of a unified object. Numerous studies have supported the idea that attention plays a crucial role in multisensory integration (for reviews, see [1,2,3,4]). A portion of these studies used EEG/ERP techniques to reveal the electrophysiological bases of these effects [5,6,7]. EEG (electroencephalogram) signals are the electrical activities of human brain that measure field potential in the space around neurons and can be categorized into several types of activities with the characteristic of specific signal frequencies and amplitudes such as: Alpha, Beta, Delta, Gamma, Theta and Mu [8]. However, it is worth mentioning that the neural responses associated with specific sensory, cognitive, and motor events are drowned within the EEG. These specific neural responses are called event-related potentials (ERPs) which can be extracted from the overall EEG by means of a simple averaging technique [9]. One of the most striking neural examples illustrating the crucial role of attention on multisensory integration is that visual attention effect can spread to the task-irrelevant auditory modality, resulting in the originally unattended auditory features of an audiovisual object being pulled into the attentional spotlight and bestowed with enhanced processing [10]. This cross-modal attentional spreading effect is typically manifested as a sustained ERP difference analogous to the attention-related auditory Nd component [11,12,13,14]. The Nd is an ERP difference component manifested as greater negative amplitude over the fronto-central scalp elicited by attended relative to unattended auditory stimuli, beginning around 150–200 ms post-stimulus [15], which is generally thought to reflect an enhancement of auditory processing due to selective attention [16,17].

The cross-modal spread of attention can be elicited either in a stimulus-driven manner [10,11,14,18,19,20] or/and in a representation-driven manner [12,13,21,22]. Specifically, the *stimulus-driven* spread of attention occurs whenever a task-irrelevant sound is presented simultaneously with an attended visual stimulus, and thus was interpreted as a bottom-up audiovisual binding process on the basis of the temporal co-occurrence of visual and auditory stimuli [13,14]. In contrast, the *representation-driven* spread of attention occurs only when the object representation of a task-irrelevant sound (e.g., a bark of a dog) is semantically congruent with that of the visual stimulus receiving extra representation-based selective attention (e.g., when searching for an image of a dog), hence was considered to be a top-down audiovisual priming process relying on the activation of highly learned associations between features in different modalities [12,13]. In addition, if a semantically congruent sound is delivered synchronously with the visual stimulus receiving representation-based selective attention, both types of the attentional spreading occur in an additive manner [8,16,17].

The stimulus-driven cross-sensory attentional spreading has been investigated more often than the representation-driven one (stimulus-driven: [10,11,14,18,19,20]; representation-driven: [12]; both: [13,21,22]), partially because the bottom-up nature of the former enables it to occur under much more circumstances. Among studies involving the stimulus-driven attentional spreading, the most intensively explored issue is the influence of higher-level audiovisual congruency (congruent vs. incongruent), but the results are mixed: some studies found a null effect [13,21] while other studies showed that its magnitude was greater in response to incongruent than congruent audiovisual pairs [18,19]. Regardless of the discrepancy, it is noteworthy that all of these studies have only manipulated the *semantic* congruency between non-emotional visual and auditory stimuli (e.g., an image of a dog with a bark of a dog vs. an image of a car with a bark of a dog). However, real-life visual and auditory stimuli may also convey emotional information (such as when seeing a championship trophy or hearing a gloomy melody), and the ability to integrate emotional information appropriately from the visual and auditory modalities is fundamental to emotion recognition and social interaction (for review, see [23]). Therefore, in order to advance our understanding regarding whether and how higher-level audiovisual congruency modulates the stimulus-driven cross-sensory spread of attention, it is necessary to take the *emotional* congruency between visual and auditory stimuli into consideration.

Human ERP studies in recent years have consistently reported that emotionally incongruent audiovisual pairs can evoke smaller auditory P2 and/or greater auditory N2 amplitudes over the fronto-central scalp than emotionally congruent audiovisual pairs [24,25,26,27,28], with the timing and scalp distribution of this P2/N2 modulation resembling the neural correlate of the stimulus-driven spread of attention (i.e., the auditory Nd difference component). Moreover, some of these studies even found an earlier occurring audiovisual emotional congruency effect during the auditory N1 interval [24,25,28]. However, it should be noted that the task paradigms utilized in these studies required participants either to *attend to the auditory modality voluntarily* and/or to *respond based on emotional information conveyed by the stimuli*. In the former case, it is insufficient to determine whether the observed P2/N2 modulation, as well as the earlier N1 modulation, is associated with the stimulus-driven attentional spreading, because the stimulus-driven process is measurable only when the crucial auditory stimuli are initially ignored, but not attended [11]. In the latter case, the task-relevant nature of high-order representations (i.e., emotions) might have rendered the underlying bottom-up, stimulus-driven attentional spreading impure if measured, which could also explain, at least in part, the aforementioned mixed findings concerning the influence of audiovisual semantic congruency (see the preceding paragraph). Therefore, in order to precisely quantify the stimulus-driven attentional spreading process and determine the effect of audiovisual emotional congruency on it, a novel experimental paradigm is needed.

**Figure 1:**
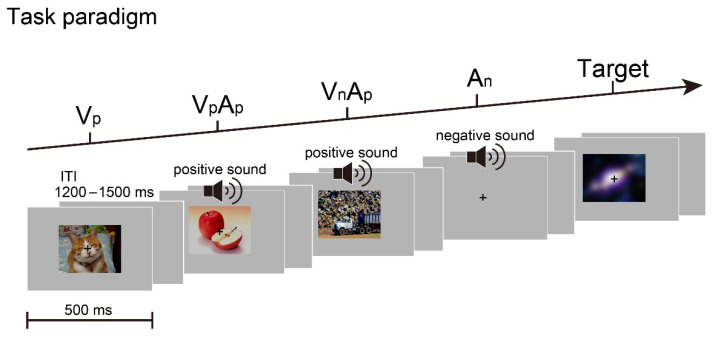
Task paradigm shown for four non-target trials and one target trial. A ***non-target*** trial could be an emotionally positive or negative visual or auditory stimulus presented alone [e.g., a positive picture (V_p_) or a negative sound (A_n_)], or the two stimuli presented synchronously (VA) to form an audiovisual pair being either emotionally congruent [e.g., a positive picture paired with a positive sound (V_p_A_p_)] or emotionally incongruent [e.g., a negative picture paired with a positive sound (V_n_A_p_)], resulting in eight sub-types of non-target stimuli. A ***target*** trial could be a blurred picture presented alone or presented synchronously with an emotionally positive or negative sound, resulting in three sub-types of target stimuli. The task for participants was to press a button in response to the target stimuli, while ignoring all sounds if delivered. Each type of trial consisted of a 500 ms stimulus presentation and an inter-trial interval (ITI) of 1200–1500 ms.

The current study investigated whether and how audiovisual emotional congruency would modulate the stimulus-driven cross-sensory spread of attention by recording ERPs in a sound-accompanying visual oddball task where emotion information was task-irrelevant and the auditory modality was unattended as well. Specifically, although emotionally positive/negative visual-only and auditory-only stimuli, as well as emotionally congruent and incongruent audiovisual pairs, were presented in the task, participants were only required to detect the rarely presented blurred pictures while ignoring all auditory stimuli if delivered (Figure 1). Our results demonstrated that the isolating auditory Nd difference component within 200–300 ms post-stimulus was greater for emotionally incongruent than congruent audiovisual stimuli when their visual constituents were emotionally negative, thereby demonstrating for the first time that audiovisual emotional congruency can modulate the stimulus-driven visual-to-auditory attentional spreading at its early phase even when emotion is task-irrelevant.

## 2. Materials and Methods

### 2.1. Subjects

Thirty volunteers participated in the assessment experiment (mean age 21.27 ± 1.29 years; 10 males and 20 females). In addition, we calculated the sample size for the formal experiment using MorePower 6.0.4 software (Saskatoon, Canada) [29]. Based on previous relevant studies, a priori sample size of 18 participants was derived by setting the parameters with *α* = 0.05, *power* = 0.80 and *η^2^_p_* = 0.119 [22]. A total of 30 participants were recruited in the formal experiment. Three participants were excluded because of excessive artifacts in EEG recordings. Data from the remaining 27 participants (mean age 21.94 ± 1.67 years; 8 males and 19 females) were included in future analysis. All participants reported normal or corrected-to-normal vision as well as normal hearing and no history of psychiatric or neurological disorders. They were naive as to the purpose of the experiment. Prior to the experiment procedures, all participants received informed consent, which was in agreement with the Declaration of Helsinki. After the experiment, participants were paid for their participation.

### 2.2. Assessment Experiment

The objective of the assessment experiment was to evaluate and standardize the emotional picture and sound materials that would be used in our formal experiment. A total of 60 pictures with 30 of positive valence and 30 of negative valence were selected through the Chinese Affective Picture System (CAPS) [30] adapted from the International Affective Picture System (IAPS) [31]. The brightness, saturation and coloration of each pixel point in the pictures were determined using the function *rgb2hsv* in Matlab. The software Adobe Photoshop CC 2019 was used to adjust the brightness, saturation and coloration of these pictures based on the average of all pixels in each picture, as recommended in the previous literature [32,33], so that each of the three parameters was comparable among pictures. All pictures were re-sized to a uniform size of 400 × 300 pixels. A total of 60 segments of voices with 30 of positive valence and 30 of negative valence were selected from the International Affective Digitized Sounds (IADS) [34] and then standardized to 500 ms in duration using the software Sound Engine, in order to meet the presentation requirement of the paradigm. The voice sampling rate was 48,000 Hz/s and all voices were tuned to a consistent volume of 65 dB SPL using the software MP3 Gain GUI. As the fundamental frequency of auditory stimuli could influence the ERP responses, a custom script in Matlab was used to measure the acoustic parameters of the auditory stimuli, including f0, pitch height, pitch range and pitch variance for each voice. Each acoustic parameter was compared between the emotionally positive and negative voices using independent-samples *t*-tests. The results showed that none of the acoustic parameters differed significantly between positive vs. negative voices [f0: *t*_(58)_ = 1.21, *p* = 0.23, *d* = 0.31; pitch height: *t*_(58)_ = −0.93, *p* = 0.36, *d* = −0.24; pitch range: *t*_(58)_ = −1.16, *p* = 0.25, *d* = −0.30; pitch variance: *t*_(58)_ = 0.02, *p* = 0.98, *d* = 0.01].

The program of the assessment experiment was scripted by Psychopy 3.0 (Python version 3.7, Nottingham, UK) [35] to present the emotional stimuli and record the participants’ responses. On each trial, a fixation was first displayed in the center of the screen for 1000 ms, followed by a picture or sound presented for 2000 ms, and then the valence and arousal of this stimulus needed to be rated on a 9-point scale, respectively, using thumbnails and the Self-Assessment Manikin (SAM) scale [36,37]. The pictures and voices were assessed separately in two sessions, with the order of precedence being counterbalanced between participants. The rating scores of valence and arousal were compared between the emotionally positive and negative stimuli using paired-samples *t*-tests, separately for pictures and voices. The results showed that there were significant valence differences between positive vs. negative pictures [*t*_(29)_ = 34.11, *p* < 0.0001, *d* = 6.23] and between positive vs. negative voices [*t*_(29)_ = 19.05, *p* < 0.0001, *d* = 3.48], such that the rated valence scores of positive stimuli [pictures: 6.74 ± 0.09 (*M* ± *SE*); voices: 6.50 ± 0.11] were higher than those of negative stimuli (pictures: 2.93 ± 0.08; voices: 3.59 ± 0.08). In contrast, neither pictures [*t*_(29)_ = −2.02, *p* = 0.053, *d* = −0.37] nor voices [*t*_(29)_ = −0.36, *p* = 0.72, *d* = −0.07] showed a significant difference between the arousal scores of positive emotion (pictures: 5.78 ± 0.11; voices: 4.94 ± 0.20) and negative emotion (pictures: 5.99 ± 0.09; voices: 5.02 ± 0.11).

### 2.3. Experimental Materials and Procedures

In the formal experiment, participants sat in a dark and sound-attenuated room with a viewing distance of approximately 80 cm away from a 27-inch LCD monitor (ASUS PG279Q, 1920 × 1080, 120 Hz) where visual stimuli were presented. Two speakers (HiVi X3) were located on either side of the monitor at an equal height parallel to the center of the monitor screen for sound presentation, so that the sound played from both speakers simultaneously would be perceived as coming from the center of the monitor [38]. During the experiment, the monitor screen remained gray (RGB: 128, 128, 128) at all times and participants were asked to maintain their eyes fixated on a black cross (RGB: 0, 0, 0; 0.3° × 0.3° in size) at the center of the screen. “Presentation” software (version 18.0, NeuroBehavioral Systems, Inc., Berkeley, CA, USA) was used to display all visual and auditory stimuli.

For the purpose of isolating the stimulus-driven cross-modal spread of attention effect when analyzing EEG data [13,14], two main types of trials, namely, non-target trial and target trial, were designed in the formal experiment. A ***non-target*** trial could be an emotional visual or auditory stimulus presented alone, or could be the two stimuli presented synchronously. The emotional visual stimulus could be one of the sixty emotional pictures selected from the assessment experiment (thirty positive pictures and thirty negative pictures; each 9.5° × 7.2° in size) equiprobably, which was presented for 500 ms at the center of the monitor (Figure 1). The emotional auditory stimulus that was also centrally presented could be one of the sixty emotional sounds (thirty positive sounds and thirty negative sounds) with equal probability, which was also 500 ms in duration (with 10 ms rise and fall periods) and was approximately 65 dB SPL at participants’ ears. These emotional pictures and sounds were presented either alone or synchronously, resulting in three main stimulus types for non-target trials [i.e., visual alone (labeled as V condition), auditory alone (A condition), and audiovisual (labeled as VA condition)]. Of note, depending on the emotional valence, there were two sub-types for V and A conditions, respectively, namely, positive visual-alone (labeled as **V_p_**), negative visual-alone (labeled as **V_n_**), positive auditory-alone (labeled as **A_p_**), and negative auditory-alone (labeled as **A_n_**). Accordingly, for VA condition, when both the visual and auditory constituents of an audiovisual pair were the same emotional valence (i.e., emotionally congruent), there were two sub-types, namely, a positive picture paired with a positive sound (labeled as **V_p_A_p_**) and a negative picture paired with a negative sound (labeled as **V_n_A_n_**). Similarly, when the visual and auditory constituents of an audiovisual pair were different in emotional valence (i.e., emotionally incongruent), there were also two sub-types, namely, a positive picture paired with a negative sound (labeled as **V_p_A_n_**) and a negative picture paired with a positive sound (labeled as **V_n_A_p_**).

Apart from the aforementioned eight sub-types of non-target trials, there were also three sub-types of the ***target*** trials. Specifically, a target trial could be a blurred picture presented alone (labeled as **T** condition), or could be a blurred picture presented synchronously with either an emotionally positive sound (labeled as **TA_p_** condition) or an emotionally negative sound (labeled as **TA_n_** condition). On a given target trial, the blurred picture could be one of the above-mentioned 60 emotional pictures with equal probability, but a Gaussian blur with a radius of 22.5 pixel was applied to it in order to render its emotional valence unrecognizable. Taken together, the eight sub-types of non-target stimuli and the three sub-types of target stimuli accounted for a total of 91.67% of the trials, with 8.33% for each sub-type. The remaining 8.33% of the trials were “blank” trials on which neither visual nor auditory stimuli were presented (labeled as **B** condition), serving as an estimation of anticipatory ERPs elicited by the expectation of upcoming stimulus (for details, see Data analysis section). The aforementioned 12 types of trials were presented in a pseudo-randomized order with an inter-trial interval (ITI) varying from 1200 to 1500 ms randomly (Figure 1). The task for participants was to press the button “J” on a keyboard with their right index finger whenever they detected a blurred picture (i.e., a target trial) while ignoring all auditory stimuli. Therefore, the novel task design here meant *not only* that the auditory stimuli were task-irrelevant (i.e., only the visual stimuli were to-be-attended), *but also* that the emotional valences of both visual and auditory stimuli were task-irrelevant. The whole experiment comprised a total of 1800 trials, which were divided into 25 blocks to complete. The duration of the whole experiment was around 90 min, and a 10-sec rest time was imposed between blocks, after which participants could continue to rest or start the next block.

### 2.4. Electrophysiological Recording and Preprocessing

Electroencephalographic (EEG) signals were continuously recorded with a NeuroScan SynAmp amplifier (NeuroScan, Inc., El Paso, TX, USA) and a custom-built 64-electrode elastic cap on which the electrodes were positioned in accordance with a modified 10-10 system montage (for details, see [39]). Two additional electrodes, AFz and M1 (left mastoid), served as the ground and reference electrodes, respectively, for online EEG recording. The horizontal electrooculogram (HEOG) was recorded using bipolar electrodes placed on the left and right outer canthi. To monitor blinks and vertical eye movements (vertical electrooculogram, VEOG), bipolar electrodes were placed above and below the participants’ left eye. The impedance of all electrodes was maintained below 5 kΩ. The digital sampling rate was 1000 Hz, and a band-pass filtering of 0.05–100 Hz was applied to the online instantaneous EEG data. All EEG and EOG data were recorded via Scan software (version 4.5, NeuroScan, Inc., El Paso, TX, USA).

For the offline preprocessing, the raw continuous EEG data were firstly down-sampled to 500 Hz and then low-pass filtered (half-amplitude cutoff = 33.75 Hz, transition band width = 7.5 Hz) to attenuate high-frequency noise arising from muscle activity and external electrical sources. The arithmetic mean of the bilateral mastoids (M1, M2) served as the re-reference for the filtered data. The re-referenced data were segmented into 800-ms epochs, which were time-locked to the onset of emotional stimulus with a 200-ms pre-stimulus baseline correction. The epochs contaminated by eye movements, eye blinks and muscle activity were then removed by automatic artifact rejection based on a threshold of ± 75 μV for both EEG and EOG electrodes. In addition, in order to prevent the interference of motor responses with EEG data, all target trials (i.e., trials on which the blurred pictures were presented) and all false alarm trials were further removed. The remaining artifact-free EEG epochs were averaged separately for each experimental condition (i.e., V_p_, V_n_, A_p_, A_n_, V_p_A_p_, V_p_A_n_, V_n_A_n_, V_n_A_p_ and B). EEG preprocessing was performed using the EEGLAB toolbox [40] and a custom script in Matlab, and subsequent ERP analysis was performed in ERPLAB [41].

### 2.5. Data Analysis

According to the previous literature on the method of isolating the stimulus-driven spread of attention [13,14], firstly, the auditory ERPs in the context of attended visual stimuli were extracted by subtracting ERPs to the unisensory visual stimuli from ERPs to the audiovisual stimuli, separately for each audiovisual emotional combination (i.e., V_p_A_p_ − V_p_; V_p_A_n_ − V_p_; V_n_A_n_ − V_n_; V_n_A_p_ − V_n_). The resulting difference waves consisted of the contributions from not only the auditory constituents of the audiovisual stimuli but also the potential cross-modal attentional spreading. Secondly, the time-locked ERPs recorded on the blank trials were subtracted from ERPs elicited by the unisensory auditory stimuli alone, separately for emotionally positive and negative sounds (i.e., A_p_ − B; A_n_ − B), in order to cancel out any pre-stimulus anticipatory activities (e.g., CNV) [42] common to all stimuli. Otherwise, these common activities would be balanced out in the extracted auditory ERPs to audiovisual stimuli but left in the ERPs elicited by auditory stimuli alone. In other words, the ERPs recorded on blank trials were used as an estimation of the pre-stimulus anticipatory ERPs [7]. Finally, the extracted auditory ERPs to audiovisual stimuli were compared with the unisensory auditory ERPs based on the auditory emotional valence [i.e., (V_p_A_p_ − V_p_) vs. (A_p_ − B); (V_p_A_n_ − V_p_) vs. (A_n_ − B); (V_n_A_n_ − V_n_) vs. (A_n_ − B); (V_n_A_p_ − V_n_) vs. (A_p_ − B)], and the differences revealed in these comparisons thus represented the stimulus-driven spread of attention effects under different audiovisual emotional combinations.

The stimulus-driven spread of attention effect was further quantified by the mean amplitude of the auditory negative difference (Nd) component, which was measured with two 100-ms time windows during 200–400 ms after the onset of emotional stimuli over six adjacent fronto-central electrodes (FC1, FCz, FC2, C1, Cz, C2). These time windows and electrodes were selected because the stimulus-driven Nd amplitude is typically maximal over there [10,13,14,21,22]. In addition, since several prior studies [24,25,28] even found an audiovisual emotional congruency effect during the auditory N1 interval (although the auditory modality was not task-irrelevant), we speculated that the stimulus-driven spread of attention in response to emotional audiovisual stimuli may occur at earlier stages of processing than that to non-emotional audiovisual stimuli. Indeed, based on visual inspection, we found that there may be a difference between the extracted auditory ERPs to audiovisual stimuli and ERPs to the unisensory auditory stimuli during the time window of the auditory N1 component. Accordingly, we also analyzed the auditory N1 component, whose mean amplitude was measured during 90–130 ms over six adjacent fronto-central electrodes (FC1, FCz, FC2, C1, Cz, C2), where its amplitude was greatest when ERP waveforms were collapsed across all conditions to be compared.

For statistical analyses, to explore whether the stimulus-driven spread of attention (i.e., the auditory Nd difference component) occurred significantly under all audiovisual emotional combinations, we conducted paired-samples *t*-tests on the mean amplitudes during the two Nd intervals between the extracted auditory ERPs to audiovisual stimuli vs. the ERPs to unisensory auditory stimuli separately for each audiovisual emotional combination [i.e., for congruent audiovisual pairs with emotionally positive visual and auditory constituents: (V_p_A_p_ − V_p_) vs. (A_p_ − B); for incongruent audiovisual pairs with emotionally positive visual and negative auditory constituents: (V_p_A_n_ − V_p_) vs. (A_n_ − B); for congruent audiovisual pairs with emotionally negative visual and auditory constituents: (V_n_A_n_ − V_n_) vs. (A_n_ − B); for incongruent audiovisual pairs with emotionally negative visual and positive auditory constituents: (V_n_A_p_ − V_n_) vs. (A_p_ − B)]. Note that we did not conduct a multi-factor repeated-measures ANOVA prior to these paired *t*-test (the same below), because these *t*-tests alone are sufficient to answer the research question above and are more straightforward, which can reduce the total number of statistical tests conducted, thereby controlling the overall Type I error rate [43]. Moreover, to verify whether the attentional spreading occurred earlier, similar paired-samples *t*-tests were conducted on the auditory N1 amplitude. In order to further examine whether the magnitude of the cross-modal attentional spreading would be modulated by audiovisual emotional congruency, additional paired-samples *t*-tests were performed on the attentional spreading effects (measured as the extracted auditory minus auditory-only ERP differences) between emotionally congruent vs. incongruent audiovisual pairs. These congruent vs. incongruent contrasts were conducted only within the time window wherein the attentional spreading effect was significant under all audiovisual pairs, and were performed separately for: (1) audiovisual pairs with positive visual constituents [congruent: (V_p_A_p_ − V_p_) − (A_p_ − B) vs. incongruent: (V_p_A_n_ − V_p_) − (A_n_ − B)] and negative visual constituents [congruent: (V_n_A_n_ − V_n_) − (A_n_ − B) vs. incongruent: (V_n_A_p_ − V_n_) − (A_p_ − B)]; and for: (2) audiovisual pairs with positive auditory constituents [congruent: (V_p_A_p_ − V_p_) − (A_p_ − B) vs. incongruent: (V_n_A_p_ − V_n_) − (A_p_ − B)] and negative auditory constituents [congruent: (V_n_A_n_ − V_n_) − (A_n_ − B) vs. incongruent: (V_p_A_n_ − V_p_) − (A_n_ − B)].

In addition, to verify our conjecture that emotionally positive stimuli would capture more attention than negative stimuli when emotion is task-irrelevant (for details, see Discussion section), we further compared the visual N1 component elicited by emotionally positive vs. negative visual stimuli (i.e., V_p_ vs. V_n_) using a paired-samples *t*-test. The visual N1 component was measured as mean amplitude within the time window of 145–175 ms over two bilaterally occipital electrodes (PO7, PO8), where its negative-going amplitude was greatest when ERP waveforms were collapsed across the two aforementioned unisensory visual conditions.

Based on the results of the traditional statistical method mentioned above, we also expected to explore the additional potential factors (e.g., subject characteristics) to explain more error variance. Hence, the mixed effects models with subjects being entered as a random effect factor were conducted for exploratory analysis (for details, see Supplementary Materials).

## 3. Results

### 3.1. Behavior Results

A one-way repeated-measures ANOVA was performed for response times (RTs) and hit rates (HRs), separately, with the factor of target type [TA_p_ (visual targets accompanied by emotionally positive sounds), TA_n_ (visual targets accompanied by negative sounds), T (visual targets alone)]. For both RTs and hit rates, there was no significant difference among different target types [RTs: TA_p_, 458.25 ± 11.08 ms (*M ± SE*); TA_n_, 459.71 ± 10.01 ms; T, 458.53 ± 9.64 ms; *F*_(2, 52)_ = 0.03, *p* = 0.95, *η^2^_p_* = 0.001; HRs: TA_p_, 99.43 ± 0.16%; TA_n_, 97.91 ± 0.85%; T, 99.19 ± 0.31%; *F*_(2, 52)_ = 3.57, *p* = 0.063 (Greenhouse–Geisser corrected due to violation of the sphericity assumption), *η^2^_p_* = 0.12]. These behavioral results indicated that the emotional sounds did not interfere substantially with the detection of the target, suggesting that the auditory stimuli were ignored as required.

### 3.2. EEG Results

#### 3.2.1. The Stimulus-Driven Spread of Attention Is Modulated by Audiovisual Emotional Congruency

**Figure 2:**
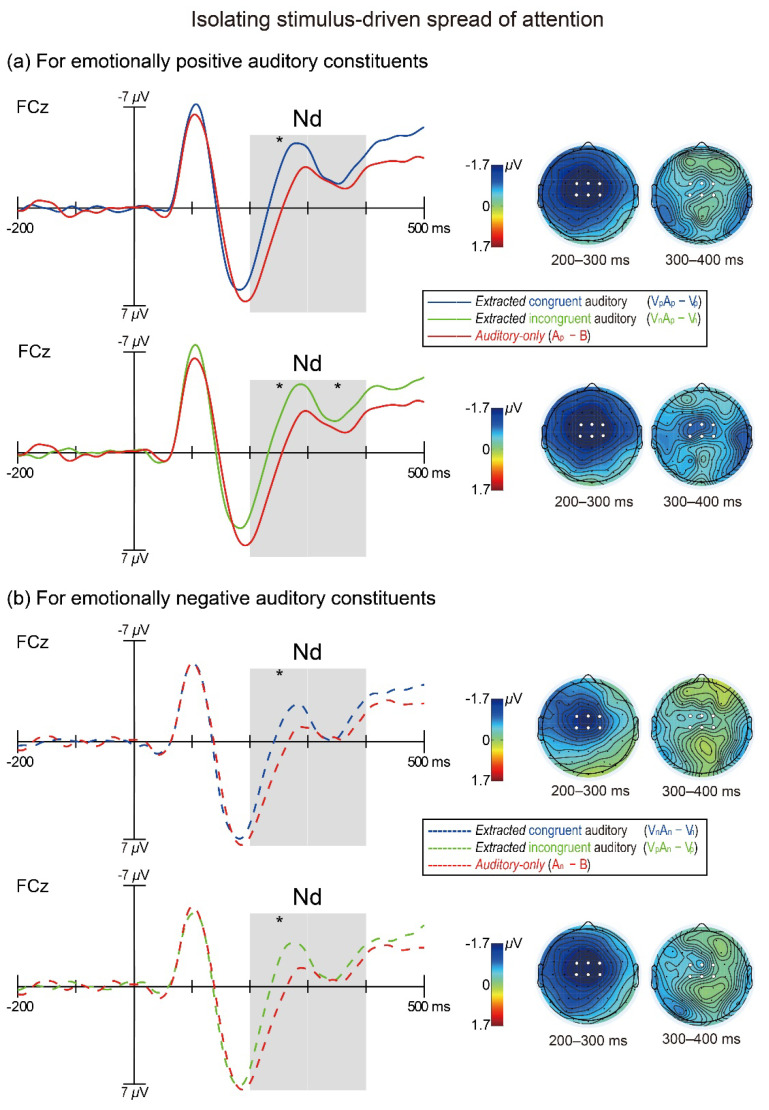
The extracted auditory ERP waveforms to emotionally congruent (blue traces) and incongruent (green traces) nontarget audiovisual stimuli and ERP waveforms evoked by auditory-only nontarget stimuli (red traces), plotted separately for (**a**) emotionally positive auditory constituents (solid traces) and (**b**) emotionally negative auditory constituents (dashed traces). These ERP waveforms are from the fronto-central electrode FCz and the shaded areas on waveforms depict the two-time windows (200–300 ms and 300–400 ms) within which the Nd component was measured. Scalp topographies are shown for the extracted-auditory minus auditory-only mean difference amplitudes during each Nd interval. The white dots on topographies depict the fronto-central ROI (FC1, FCz, FC2, C1, Cz, C2) over which the Nd component was measured. The stimulus-driven Nd component, indexed by significantly greater negative amplitude in the extracted auditory than auditory-only waveform, was prominent for all audiovisual emotional combinations within the time window of 200–300 ms. In contrast, only the incongruent audiovisual pairs with emotionally positive auditory and negative visual constituents (V_n_A_p_) produced a sustained Nd within the time window of 300–400 ms. *: *p* < 0.05 for the extracted-auditory vs. auditory-only contrast.

To investigate whether the non-target emotional stimuli elicited the stimulus-driven spread of attention as well as its time course, paired-samples *t*-tests were conducted on the mean amplitudes during each Nd interval (200–300 ms, 300–400 ms) between the extracted auditory ERPs to audiovisual stimuli (VA − V) vs. the ERPs to auditory-only stimuli (A − B) separately for each audiovisual emotional combination. A significant difference would reveal that the auditory Nd component was prominent and the stimulus-driven attentional spreading occurred reliably. The results showed that in the time window of 200–300 ms, the auditory Nd component was prominent in response to all audiovisual emotional combinations. Specifically, for the audiovisual pairs with emotionally positive auditory constituents (Figure 2a), both the extracted auditory ERPs to emotionally congruent audiovisual stimuli [V_p_A_p_ −V_p_: −1.29 *±* 0.53 μV (*M ± SE*)] and the extracted auditory ERPs to incongruent audiovisual stimuli (V_n_A_p_ − V_n_: −1.50 *±* 0.51 μV) were significantly more negative than the ERPs to auditory-only stimuli [A_p_ − B: 1.05 *±* 0.46 μV; *t*_(26)_ = −3.50, *p* = 0.002, *d* = −0.67; *t*_(26)_ = −3.87, *p* < 0.001, *d* = −0.75). Similarly, for the audiovisual pairs with emotionally negative auditory constituents (Figure 2b), both the extracted auditory ERPs to congruent audiovisual stimuli (V_n_A_n_ − V_n_: 0.01 *±* 0.54 μV) and the extracted auditory ERPs to incongruent audiovisual stimuli (V_p_A_n_ − V_p_: −0.25 *±* 0.53 μV) were significantly more negative than the ERPs to auditory-only stimuli [A_n_ − B: 1.82 *±* 0.41 μV; *t*_(26)_ = −2.78, *p* = 0.01, *d* = −0.53; *t*_(26)_ = −2.92, *p* = 0.007, *d* = −0.55). These results indicate that the stimulus-driven spread of attention effect occurred regardless of whether the audiovisual pairs were emotionally congruent or incongruent during the time window of 200–300 ms.

In contrast, in the time window of 300–400 ms, the extracted auditory ERPs to audiovisual stimuli were found to be significantly more negative than the ERPs to auditory-only stimuli only for the incongruent audiovisual pairs with emotionally positive auditory constituents [V_n_A_p_ − V_n_: −2.79 *±* 0.44 μV; A_p_ − B: −1.90 *±* 0.38 μV; *t*_(26)_ = −2.13, *p* = 0.043, *d* = −0.41; Figure 2a], but not for the congruent audiovisual pairs with emotionally positive auditory constituents [V_p_A_p_ − V_p_: −2.39 *±* 0.52 μV; A_p_ − B: −1.90 *±* 0.38 μV; *t*_(26)_ = −1.12, *p* = 0.27, *d* = −0.22], or the incongruent audiovisual pairs with emotionally negative auditory constituents [V_p_A_n_ − V_p_: −1.13 *±* 0.46 μV; A_n_ − B: −0.72 *±* 0.41 μV; *t*_(26)_ = −0.78, *p* = 0.44, *d* = −0.15; Figure 2b], or the congruent audiovisual pairs with emotionally negative auditory constituents [V_n_A_n_ − V_n_: −1.05 *±* 0.46 μV; A_n_ − B: −0.72 *±* 0.41 μV; *t*_(26)_ = −0.60, *p* = 0.56, *d* = −0.12]. These results suggests that there might be two prerequisites for the cross-modal attentional spreading to be sustained into the 300–400 ms time window in response to emotional audiovisual stimuli, one being that the auditory constituents of audiovisual stimuli are emotionally positive (i.e., the visual constituents are emotionally negative) and the other being that there is an emotional conflict between the auditory and visual constituents. Therefore, the late phase of the stimulus-driven attentional spreading to emotional sounds can be modulated by audiovisual emotional congruency.

**Figure 3:**
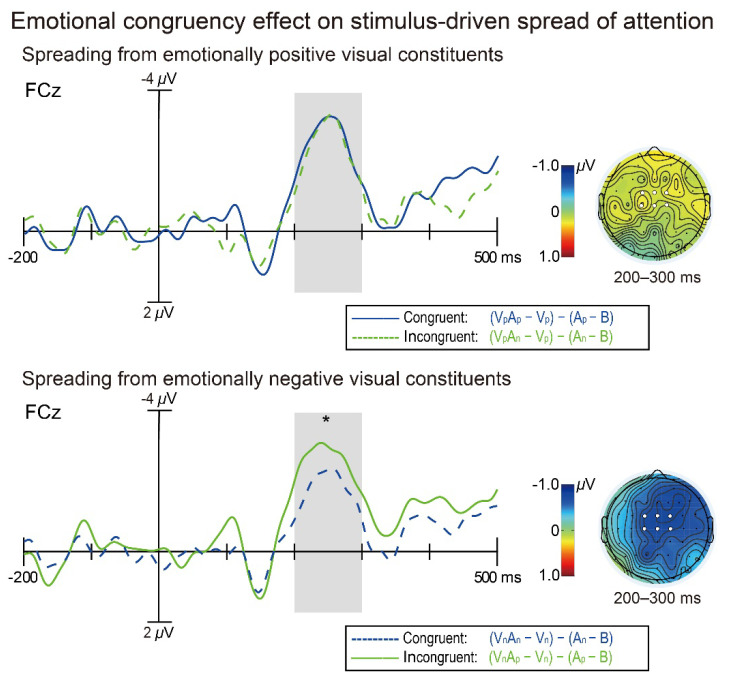
The *magnitude* of the stimulus-driven auditory Nd wave (measured as the extracted-auditory minus auditory-only ERP difference) in response to emotionally congruent (blue traces) and incongruent (green traces) audiovisual stimuli, plotted separately for audiovisual stimuli with emotionally positive (upper half) and negative (lower half) visual constituents. The shaded areas on waveforms depict the time window of 200–300 ms within which the Nd magnitudes were further contrasted. Scalp topographies are shown for incongruent minus congruent Nd magnitude differences during the time window of 200–300 ms. The Nd magnitude was significantly larger for emotionally incongruent than congruent audiovisual stimuli only when their visual constituents’ emotional valence was negative. *: *p* < 0.05 for the incongruent vs. congruent contrast.

Notably, although the early phase of the stimulus-driven attentional spreading (i.e., 200–300 ms) could *occur* regardless of audiovisual emotional congruency, it was still unclear whether the *magnitude* of the early-phase attentional spreading would be modulated by audiovisual emotional congruency. To examine this question in detail, additional paired-samples *t*-tests were performed on the attentional spreading effects (measured as the extracted auditory minus auditory-only ERP differences) during 200–300 ms between emotionally congruent vs. incongruent audiovisual pairs in the following two ways. Firstly, when these comparisons were anchored to the *visual* constituents’ emotional valence, the attentional spreading effect for emotionally incongruent audiovisual stimuli [(V_n_A_p_ − V_n_) − (A_p_ − B): −2.13 *±* 0.43 μV] was found to be significantly greater than that for congruent audiovisual stimuli [(V_n_A_n_ − V_n_) − (A_n_ − B): −1.40 *±* 0.51 μV] only when the visual constituents were emotionally negative [*t*_(26)_ = −2.33, *p* = 0.028, *d* = −0.45; Figure 3, lower half], but not when the visual constituents were emotionally negative [incongruent: (V_p_A_n_ − V_p_) − (A_n_ − B) = −1.89 *±* 0.51 μV; congruent: (V_p_A_p_ − V_p_) − (A_p_ − B) = −2.08 *±* 0.44 μV; *t*_(26)_ = 0.64, *p* = 0.53, *d* = 0.12; Figure 3, upper half]. Secondly, when similar incongruent vs. congruent comparisons were anchored to the *auditory* constituents’ emotional valence, however, we found no significant difference either when the auditory constituents were emotionally positive [incongruent: (V_n_A_p_ − V_n_) − (A_p_ − B) = −2.13 *±* 0.43 μV; congruent: (V_p_A_p_ − V_p_) − (A_p_ − B) = −2.08 *±* 0.44 μV; *t*_(26)_ = −0.20, *p* = 0.84, *d* = −0.04] or when they were emotionally negative [incongruent: (V_p_A_n_—V_p_)—(A_n_—B) = −1.89 *±* 0.51 μV; congruent: (V_n_A_n_ − V_n_) − (A_n_ − B) = −1.40 *±* 0.51 μV; *t*_(26)_ = −1.45, *p* = 0.16, *d* = −0.28]. Taken together, these findings demonstrate that audiovisual emotional congruency can modulate the early-phase stimulus-driven attentional spreading if the audiovisual pairs’ visual constituents are emotionally negative, which echoes the aforementioned finding that the late-phase (300–400 ms) attentional spreading occurred only for the incongruent pairs with emotionally negative visual constituents (Figure 2a).

#### 3.2.2. Post Hoc Exploratory Analyses

Further visual inspection of Figure 2a implies that when the audiovisual pairs’ auditory constituents were emotionally positive, the extracted auditory ERPs to both congruent and incongruent audiovisual stimuli seem larger than the ERPs to unisensory auditory stimuli during the time window of auditory N1 component, which may reflect the cross-modal attentional spreading occurring in advance for emotional audiovisual stimuli. To explore this possibility, we performed paired-samples *t*-tests on the auditory N1 amplitude (measured over 90–130 ms) between the extracted auditory ERPs to audiovisual stimuli vs. the ERPs to auditory-only stimuli separately for each of the four audiovisual emotional combinations. However, none of the four *t*-tests’ results reached statistical significance [V_p_A_p_ − V_p_: −5.75 *±* 0.57 μV; V_n_A_p_ − V_n_: −5.84 *±* 0.62 μV; A_p_ − B: −5.41 *±* 0.62 μV; (V_p_A_p_ − V_p_) vs. (A_p_ − B): *t*_(26)_ = −0.92, *p* = 0.37, *d* = −0.18; (V_n_A_p_ − V_n_) vs. (A_p_ − B): *t*_(26)_ = −1.28, *p* = 0.21, *d* = −0.25; c.f., Figure 2a; V_n_A_n_ −V_n_: −4.08 *±* 0.53 μV; V_p_A_n_ − V_p_: −3.71 *±* 0.51 μV; A_n_ − B: −4.14 *±* 0.54 μV; (V_n_A_n_ − V_n_) vs. (A_n_ − B): *t*_(26)_ = 0.23, *p* = 0.82, *d* = 0.04; (V_p_A_n_ − V_p_) vs. (A_n_ − B): *t*_(26)_ = 1.27, *p* = 0.21, *d* = 0.25; c.f., Figure 2b]. Therefore, there is no substantial evidence to propose that the stimulus-driven cross-modal spread of attention occurs earlier for emotional than non-emotional of audiovisual stimuli.

**Figure 4:**
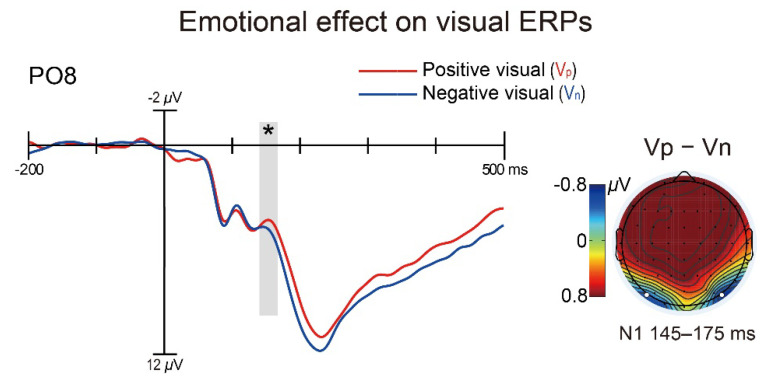
ERP waveforms elicited by emotionally positive visual-only (blue lines: V_p_) and negative visual-only (green lines: V_n_) stimuli, exemplified from the right occipital electrode PO8. The shaded area on waveforms depicts the time window of 145–175 ms within which the visual N1 component was measured. The scalp topography is shown for positive visual-only minus negative visual-only mean difference amplitudes during the visual N1 interval. The amplitude of N1 was larger for positive visual-only than negative visual-only stimuli. *: *p* < 0.05 for the contrast.

Finally, to validate our assumption that emotionally positive stimuli would capture more attention than negative stimuli when emotion is task-irrelevant (for details, see Discussion section), we conducted a paired-samples *t*-test on the visual N1 amplitude (measured within 145–175 ms over electrodes PO7 and PO8) between emotionally positive and negative unisensory visual stimuli that were nontargets but spatially attended. The result showed that the N1 component evoked by positive visual stimuli (V_p_: 4.17 *±* 3.91 μV) was more negative-going in amplitude than that evoked by negative visual stimuli [V_n_: 4.76 *±* 3.80 μV; *t*_(26)_ = −2.23, *p* = 0.035, *d* = −0.43; see Figure 4]. This finding implies that when the emotions of attended visual stimuli are irrelevant to the current task, the emotionally positive ones would capture more attention than the negative ones, thereby providing evidence for the assumption mentioned above.

## 4. Discussion

The current study utilized the high time-resolution ERP technique to explore whether the stimulus-driven attentional spreading from an audiovisual pair’s attended visual constituent to its unattended auditory constituent [10] would be affected by high-level emotional congruency between the visual and auditory constituents. In order to render the visual constituents attended and the auditory constituents unattended, while keeping emotion information carried by all stimuli task-irrelevant, we required participants to only detect the rarely presented blurred pictures (targets) under the premise of ignoring all sounds, although emotionally positive/negative visual-only and auditory-only stimuli, as well as emotionally congruent and incongruent audiovisual pairs, were presented (as nontargets) in the task. Our behavioral data showed that the target detection performance, quantified by both RTs and hit rates, was neither improved nor impaired when the blurred pictures were paired with emotionally positive sounds (TA_p_) or negative sounds (TA_n_), relative to when the blurred pictures were presented alone (T). The absence of previously reported behavioral modulations of emotional sounds [44] suggest that our participants ignored the task-irrelevant auditory inputs as well as emotion information to a high degree as required.

Our electrophysiological data first yielded that the auditory Nd component, indexed by significantly greater negative amplitude in the extracted auditory ERPs to emotional audiovisual stimuli (e.g., V_n_A_p_ − V_n_) than in the ERPs to emotional auditory-only stimuli (e.g., A_p_ − B), was prominent for all audiovisual emotional combinations (i.e., V_p_A_p_, V_n_A_n_, V_p_A_n_ and V_n_A_p_) within the time window of 200–300 ms. These findings indicate that the *occurrence* of stimulus-driven visual-to-auditory attentional spreading is independent of audiovisual emotional congruency, confirming its bottom-up nature as proposed in previous studies [13,14,18,20]. However, the more important finding is that the amplitude of Nd component within the 200–300 ms interval, measured as the extracted-auditory minus auditory-only ERP difference, was significantly greater in response to emotionally incongruent than congruent audiovisual pairs when their visual constituents were emotionally negative. Furthermore, the Nd component was found to extend into 300–400 ms only in response to the incongruent audiovisual stimuli with emotionally negative visual constituents. Given that the task-irrelevance of high-level emotional representations in the current paradigm avoided the potential top-down contamination when quantifying the bottom-up, stimulus-driven Nd component, the findings above provide strong and convergent evidence that audiovisual emotional congruency does have a substantial influence on the *magnitude* of stimulus-driven cross-modal spread of attention, beginning in parallel with its occurrence.

It is noteworthy that the auditory Nd amplitude was larger for emotionally incongruent than congruent audiovisual pairs only when their visual constituents were emotionally negative (i.e., V_n_A_p_ vs. V_n_A_n_) but not when their visual constituents were emotionally positive (i.e., V_p_A_n_ vs. V_p_A_p_), indicating that the audiovisual emotional congruency effect is specific to the attentional spreading from visual constituents conveying negative emotions. One might interpret these findings in terms of the well-known “negativity bias” that emotionally negative stimuli, given its superior biological significance, can attract more attentional resources than emotionally positive and neutral stimuli at early stages of processing, which typically leads to greater P1 and/or N1 components in the visual domain [45,46,47,48,49]. In the current visual oddball task, this interpretation would propose that the visual elements of audiovisual pairs captured more attention when these visual elements were emotionally negative than positive, leading to the task-irrelevant auditory elements of the former being bestowed with even more enhanced processing as attention spread across modality. Consequently, the emotional conflicts in incongruent audiovisual pairs with negative visual elements might be detected more sufficiently, hence the observed “negative-visual-specific” emotional congruency effect. However, it should be noted that the basic assumption of this plausible interpretation does not hold in the current study, because our post hoc analysis has shown that the visual N1 amplitude was actually *smaller*, instead of larger, in response to negative than positive visual-only stimuli (see Figure 4), which suggests that positive pictures captured more attention than negative pictures in the current study, not the other way around.

In fact, several prior studies have also observed larger N1 amplitudes elicited by emotionally positive than negative visual stimuli [50,51], and a common feature in these studies is that the emotional characteristics of stimuli were *irrelevant* to their participants’ tasks, in contrast to those studies showing the negativity bias wherein emotions were typically task-*relevant* [45,46,48,49] (but see [47]). Since the emotional characteristics of visual stimuli were also task-irrelevant in the current study, it is possible that early attentional resources were allocated more to the current positive than negative visual stimuli (and visual constituents of audiovisual pairs). If that is the case, we should further predict that the attentional spreading from positive visual constituents was stronger than that from negative visual constituents. Indeed, this prediction is supported, at least in part, by another post hoc test yielding that the isolated auditory Nd amplitude tended to be larger for congruent audiovisual pairs with emotionally positive visual constituents than congruent pairs with negative visual constituents [*t*_(26)_ = −1.80, *p* = 0.083, *d* = −0.35; see Figure 3, blue solid and dashed traces]. After substantiating the existence of “positivity bias” in the current study, we proposed that for audiovisual pairs with emotionally negative visual constituents, as visual attention spread across modality to auditory constituents anyway, the incongruent (i.e., V_n_A_p_) pairs’ positive auditory constituents may attract further attention than the congruent (i.e., V_n_A_n_) pairs’ negative auditory constituents, thereby intensifying the implicit conflict processing of the incongruent pairs and leading to the significant audiovisual emotional congruency effect in this case. In contrast, for audiovisual pairs with emotionally positive visual constituents, the incongruent (i.e., V_p_A_n_) pairs’ negative auditory constituents may not capture extra attention relative to the congruent (i.e., V_p_A_p_) pairs’ positive auditory constituents, which could render the conflict processing of these incongruent pairs to a limited extent, resulting in the observed null effect of audiovisual emotional congruency in that case. Although the interpretation above is relatively tentative, it highlights the subtle interplay between audiovisual emotional congruency and certain emotional combinations in modulating the stimulus-driven attentional spreading, which is consistent with many previous EEG investigations showing that the effects of audiovisual emotional congruency were specific to certain emotional combinations [24,25,26,27].

It should also be noted that since we explored the audiovisual emotional congruency effect separately for bimodal pairs with positive (i.e., V_p_A_n_ vs. V_p_A_p_) and negative (i.e., V_n_A_p_ vs. V_n_A_n_) visual constituents, it is *inevitable* that the emotionally congruent and incongruent pairs differed not only in the degree of congruency, but also in overall valence. For example, for audiovisual pairs with negative visual constituents, the overall valence of the incongruent pairs (V_n_A_p_) was certainly higher than that of the congruent pairs (V_n_A_n_). Accordingly, one may argue that the current larger Nd amplitude to the incongruent than congruent pairs with negative visual constituents (Figure 3, lower half) was simply due to the overall valence of the incongruent pairs V_n_A_p_ being higher than that of the congruent pairs V_n_A_n_, rather than the implicit conflict processing of the incongruent pairs. However, note that for audiovisual pairs with positive visual constituents, the overall valence of the incongruent pairs (V_p_A_n_) was definitely *lower* than that of the congruent pairs (V_p_A_p_). Therefore, had this high-valence hypothesis alone held, the Nd amplitude to the incongruent pairs V_p_A_n_ should have been smaller than that to the congruent pairs V_p_A_p_, but was not (Figure 3, upper half). Based on the ratiocination, we argued that although the role of the audiovisual emotional pairs’ overall valence in the congruency effect on stimulus-driven attentional spreading cannot be ruled out in the current study, the implicit conflict processing of the incongruent pairs did contribute substantially to the congruency effect on attentional spreading. Nevertheless, additional research with improvements in the experimental paradigm is strongly required to tease apart the influences of emotional conflict and valence when examining the audiovisual emotional congruency effect.

The current audiovisual *emotional* congruency effect started approximately 200 ms post-stimulus, in parallel with the emergence of stimulus-driven attentional spreading. In contrast, the audiovisual *semantic* congruency effect reported in previous research on stimulus-driven attentional spreading did not begin until around 300 ms post-stimulus [18] (but see [21] for a null result). This discrepancy suggests that the emotional conflict between visual and auditory elements can be processed more rapidly than the semantic conflict between emotionally neutral visual and auditory elements. Indeed, previous electrophysiological studies have revealed that over the fronto-central scalp (i.e., auditory ROI), the timing of audiovisual emotional congruency effect (typically prior to 200 ms [24,25,26,27,28]) was earlier than that of audiovisual semantic congruency effect (starting ~250 ms at the earliest [52]) even when participants needed to actively evaluate the emotional/semantic characteristics of stimuli. However, although some of these studies [24,25,28] even reported a more rapid audiovisual emotional congruency effect during the auditory N1 interval, we did not find its counterpart when measuring the cross-modal attentional spreading, as indexed by the absence of significant difference between the extracted-auditory vs. auditory-only ERPs during the auditory N1 interval for all audiovisual pairs. One reason for this null result could be that the combined task-irrelevance of emotional information and auditory inputs led to the underlying emotional congruency effect being delayed. Additional research with a larger sample size might be needed to further confirm this null result and hence our interpretation of it.

## 5. Conclusions

In summary, the current ERP data provides clear evidence that the stimulus-driven attentional spreading from an audiovisual pair’s attended visual constituent to its unattended auditory constituent could be modulated by emotional congruency between the visual and auditory constituents even when the emotional characteristics of all stimuli were task-irrelevant. This modulation emerged at the same time as the stimulus-driven attentional spreading occurred (~200 ms post-stimulus), and was further contingent on the emotional valence (positive/negative) of the audiovisual pair’s visual constituent. These findings not only reveal when and how audiovisual emotional congruency influences the stimulus-driven cross-sensory attentional spreading in particular, adding to the existing studies focusing on the influence of audiovisual semantic congruency [13,18,19,21], but also advance our understanding regarding how high-level stimulus representation affects the low-level, bottom-up audiovisual binding process in general. The limitation of the work is that in order to ensure the task-irrelevance of emotional information (for the purpose of providing strong evidence for our findings), the *representation*-driven cross-sensory attentional spreading process, whose occurrence would require participants to selectively attend to a particular emotion in the visual modality, did not exist in the current study, hence could not be isolated and investigated in parallel [13]. Further studies with task designs such as the above might be required to concurrently examine the influence of audiovisual emotional congruency on both the stimulus- and representation-driven spreading of attention. Furthermore, recent studies have shown significant gender differences in cross-modal emotion perception [53,54]. It could also be a potential factor influencing the emotional cross-modal attentional spreading reported here. However, the insufficient number of participants recruited for each gender (8 males and 19 females) prevented us from exploring the gender difference with confidence. Further studies with larger sample sizes should examine this potential difference to uncover the role of gender in the emotional cross-modal spread of attention.

## Data Availability

The datasets presented in this article are not readily available because the datasets involve unfinished research projects. If necessary, those wishing to access the datasets should contact the corresponding authors.

## Supplementary Materials

The following supporting information can be downloaded at: https://www.mdpi.com/article/10.3390/brainsci12091229/s1, Supplementary Information: Mixed effects models. References [55,56] are cited in the Supplementary Materials.
