# Supplementary material for: Audiovisual Emotional Congruency Modulates the Stimulus-Driven Cross-Modal Spread of Attention
Source: Brain Sci. 2022 Sep 10;12(9):1229. doi: 10.3390/brainsci12091229 (PMC9497153; doi:10.3390/brainsci12091229)
Supplement: Supplementary file 1 [file brainsci-12-01229-s001.zip › brainsci-1890386-supplementary.pdf]

Supporting Information

Mixed effects models

Statistical Analyses

A series of mixed-effects models in R (Version 4.2.1) with lmeTest packages (Kuznetsova et al., 2017) was used for exploratory analysis. The method of isolating the stimulus-driven spread of attention was consistent with the description in the main text (for details, see Section 2.5). To verify whether the stimulus-driven spread of attention occurred significantly under all audiovisual emotional combinations, the mean ERP amplitudes during two Nd intervals were entered as dependent variables in the mixed-effects models. The within-subject variable stimulus type (three conditions: congruent audiovisual pairs, incongruent audiovisual pairs, and unisensory auditory) was entered as categorical fixed factors, in which the unisensory auditory condition was set as the baseline level. Note that in order to compare with our previous statistic results, we conducted two mixed-effects models separately for positive and negative sounds (i.e., for positive sounds: congruent audiovisual pairs with emotionally positive visual and auditory constituents (V_pA_p - V_p) versus incongruent audiovisual pairs with emotionally negative visual and positive auditory constituents (V_nA_p - V_n) versus unisensory positive auditory (A_p - B); for negative sounds: congruent audiovisual pairs with emotionally negative visual versus auditory constituents (V_nA_n - V_n), incongruent audiovisual pairs with emotionally positive visual and negative auditory constituents (V_pA_n - V_p) versus unisensory negative auditory (A_n - B)).

Furthermore, to examine whether the stimulus-driven spread of attention occurred at an earlier interval, similar mixed-effects models were used on the auditory N1 amplitude. To further investigate whether audiovisual emotional congruency would modulate the magnitude of the cross-modal attentional spreading, other mixed-effects models were used on the attentional spreading effects (measured as the extracted auditory minus auditory-only ERP differences) between emotionally congruent versus incongruent audiovisual pairs separately for anchoring to visual constituents or auditory constituents with different emotional valence (i.e., for positive visual constituents: congruent attentional spreading effects (V_pA_p - V_p) - (A_p - B) v.s. incongruent

attentional spreading effects (V_pA_n - V_p) - (A_n-B); for negative visual constituents: congruent attentional spreading effects (V_nA_n - V_n) - (A_n - B) v.s. incongruent attentional spreading effects (V_nA_p – V_n) - (A_p - B); for positive auditory constituents: congruent attentional spreading effects (V_pA_p - V_p) - (A_p - B) v.s. incongruent attentional spreading effects (V_nA_p – V_n) - (A_p-B); for negative auditory constituents: congruent attentional spreading effects (V_nA_n - V_n) - (A_n - B) v.s. incongruent attentional spreading effects (V_pA_n – V_p) - (A_n - B)). In this model, the mean ERP amplitudes of Nd were entered as dependent variables, and the congruency of audiovisual pairs was entered as a categorical fixed factor (two conditions: congruent audiovisual pairs and incongruent audiovisual pairs) in which the emotionally incongruent audiovisual pairs were set as the baseline level.

Finally, an additional mixed-effects model on the mean amplitude of visual N1 was used to confirm our assumption that emotionally positive stimuli would capture more attention than negative stimuli when emotion is task-irrelevant. The within-subject variable visual valence (two conditions: positive visual and negative visual) was entered as a categorical fixed factor in which positive visual was set as the baseline level. For all statistical tests above, *subjects* were entered as a random effect factor for intercepts. Tukey's post hoc tests in the emmeans package (Lenth, 2020) were implemented for pairwise comparison in case of a significant main effect.

Results

Behavior results

A mixed-effects model was performed for response times (RTs) and hit rates (HRs), separately, with the within-subject factor of target type as categorical fixed factors (TA_p (visual targets accompanied by emotionally positive sounds), TA_n (visual targets accompanied by negative sounds), T (visual targets alone)). The visual targets alone (T) was set as the baseline level, and subjects were entered as a random factor for intercepts. For RTs, there was no significant difference among different target types (RTs: $F_{(2, 52)}$ = 0.03, p = 0.97). However, mixed-effects analyses showed significant main effects of target types for hit rates ($F_{(2, 52)}$ = 3.62, p < 0.05). Compared with visual targets alone (T), the hit rate for visual targets accompanied by negative sounds (TA_n) was

significantly lower ($t_{(52)} = -2.51$, $p < 0.05$), but that for visual targets accompanied by positive sounds was not (TA_p, $t_{(52)} = -0.41$, $p = 0.69$). The hit rate for visual targets accompanied by negative sounds (TA_n) was also lower than that for visual targets accompanied by positive sounds (TA_p, $t_{(52)} = -2.10$, $p < 0.05$).

EEG results

To verify whether the non-target emotional stimuli elicited the stimulus-driven spread of attention as well as its time course after treating subjects as random factors, mixed-effects models were used on the mean amplitudes during each Nd interval (200-300 ms, 300-400 ms) separately for positive and negative auditory constituents. The mixed-effects analyses showed a significant main effect of stimulus types both for positive and negative auditory constituents in the time window of 200-300 ms (positive: $F_{(2,52)} = 13.05$, $p < 0.001$; negative: $F_{(2,52)} = 7.37$, $p < 0.01$, Figure 2). Compared with the ERPs to auditory-only stimuli (A - B), both the extracted auditory ERPs to emotionally congruent ($V_pA_p - V_p$: $t_{(52)} = -4.22$, $p < 0.001$; $V_nA_n - V_n$: $t_{(52)} = -3.08$, $p = 0.003$) and incongruent ($V_nA_p - V_p$: $t_{(52)} = -4.60$, $p < 0.001$; $V_pA_n - V_p$: $t_{(52)} = -3.52$, $p < 0.001$) audiovisual pairs were significantly more negative-going regardless of the valence of auditory constituents. Conversely, there were no significant main effects for either positive or negative auditory constituents in the time window of 300-400 ms (positive: $F_{(2,52)} = 2.59$, $p = 0.08$; negative: $F_{(2,52)} = 0.41$, $p = 0.66$, Figure 2). However, the extracted auditory ERPs to incongruent audiovisual pairs with emotionally positive auditory constituents were significantly more negative than the ERPs to auditory-only stimuli, which were treated as the contrast baseline level ($t_{(52)} = -2.27$, $p < 0.05$).

Even though the results for the occurrence of the stimulus-driven spread of attention in the time window of 300-400 ms have already illustrated the modulation of emotional congruency on attentional spreading in the late processing phase, it was still unclear whether the magnitude of the early phase attentional spreading would be modulated by audiovisual emotional congruency. To further examine this question clearly, mixed-effects models were used in the determination of attentional spreading effects (measured as the extracted auditory minus auditory-only ERP differences) during the 200-300 ms between emotionally congruent versus incongruent audiovisual

pairs in the following two ways. The first method used was to anchor to the visual constituents' emotional valence; the attentional spreading effect for emotionally incongruent audiovisual stimuli was found to be significantly greater than that for congruent audiovisual stimuli only when the visual constituents were emotionally negative ((V_nAp - V_n) - (A_p - B) v.s. (V_nA_n - V_n) - (A_n - B); $t_{(26)} = -2.33$, $p = 0.028$; Figure 3, lower half), but not when the visual constituents were emotionally positive ((V_pA_n - V_p) - (A_n - B) v.s. (V_pA_p - V_p) - (A_p - B); $t_{(26)} = 0.64$, $p = 0.53$; Figure 3, upper half). The second method used was to anchor to the auditory constituents' emotional valence. However, no significant difference was found either when the auditory constituents were emotionally positive ((V_nA_p - V_n) - (A_p - B) v.s. (V_pA_p - V_p) - (A_p - B); $t_{(26)} = -0.20$, $p = 0.84$) or when they were emotionally negative ((V_pA_n - V_p) - (A_n - B) v.s. (V_nA_n - V_n) - (A_n - B); $t_{(26)} = -1.45$, $p = 0.16$).

Due to the reason mentioned in the main text, the difference in auditory N1 amplitude should be tested by mixed-effects models as well. The results showed that there was no significant main effect of stimulus types for positive auditory constituents ((V_pA_p - V_p) v.s. (V_nA_p - V_n) v.s. (A_p - B); $F_{(2,52)} = 0.79$, $p = 0.46$) and negative auditory constituents ((V_nA_n – V_n) v.s. (V_pA_n – V_p) v.s. (A_n - B); $F_{(2,52)} = 1.04$, $p = 0.36$).

Finally, to validate our assumption that emotionally positive stimuli would capture more attention than negative stimuli when emotion is task-irrelevant, the mixed-effects model was used to determine the visual N1 amplitude between emotionally positive and negative unisensory visual stimuli that were nontargets but spatially attended. Compared to the negative unisensory visual stimuli, the visual N1 amplitude to positive visual stimuli was more negative ($t_{(26)} = -2.23$, $p = 0.035$; see Figure 4).

Discussion

As the above statistical results showed, the mixed-effects models still yielded similar results as the traditional statistical approach. Although the *subjects* that were used as a random factor explained a portion of the variance of residuals, our main results were not influenced by this approach. Thus, subject characteristics are not a potential factor influencing the cross-modal attentional spreading. However, the results of hit rates have changed. We found that the hit rates of visual targets accompanied by

negative sounds (TA_n) were significantly lower than visual targets accompanied by positive sounds (TA_n) and visual targets alone (T), which indicated that the differences in subjects' characteristics only affect the responses to visual targets accompanied by negative sounds (TA_n).

References

Kuznetsova, A., Brockhoff, P. B., & Christensen, R. H. (2017). lmerTest package: tests in linear mixed effects models. *Journal of statistical software*, 82, 1-26.

Lenth, R. (2020). Emmeans: Estimated marginal means, aka leastsquares means. R package
